# Inhibition of Post-Transcriptional RNA Processing by CDK Inhibitors and Its Implication in Anti-Viral Therapy

**DOI:** 10.1371/journal.pone.0089228

**Published:** 2014-02-21

**Authors:** Jitka Holcakova, Petr Muller, Peter Tomasec, Roman Hrstka, Marta Nekulova, Vladimir Krystof, Miroslav Strnad, Gavin W. G. Wilkinson, Borivoj Vojtesek

**Affiliations:** 1 Regional Centre for Applied Molecular Oncology, Masaryk Memorial Cancer Institute, Brno, Czech Republic; 2 School of Medicine, Cardiff University, Cardiff, United Kingdom; 3 Laboratory of Growth Regulators, Faculty of Science, Palacky University, Olomouc, Czech Republic; 4 Institute of Experimental Botany AS CR, Olomouc, Czech Republic; George Mason University, United States of America

## Abstract

Cyclin-dependent kinases (CDKs) are key regulators of the cell cycle and RNA polymerase II mediated transcription. Several pharmacological CDK inhibitors are currently in clinical trials as potential cancer therapeutics and some of them also exhibit antiviral effects. Olomoucine II and roscovitine, purine-based inhibitors of CDKs, were described as effective antiviral agents that inhibit replication of a broad range of wild type human viruses. Olomoucine II and roscovitine show high selectivity for CDK7 and CDK9, with important functions in the regulation of RNA polymerase II transcription. RNA polymerase II is necessary for viral transcription and following replication in cells. We analyzed the effect of inhibition of CDKs by olomoucine II on gene expression from viral promoters and compared its effect to widely-used roscovitine. We found that both roscovitine and olomoucine II blocked the phosphorylation of RNA polymerase II C-terminal domain. However the repression of genes regulated by viral promoters was strongly dependent on gene localization. Both roscovitine and olomoucine II inhibited expression only when the viral promoter was not integrated into chromosomal DNA. In contrast, treatment of cells with genome-integrated viral promoters increased their expression even though there was decreased phosphorylation of the C-terminal domain of RNA polymerase II. To define the mechanism responsible for decreased gene expression after pharmacological CDK inhibitor treatment, the level of mRNA transcription from extrachromosomal DNA was determined. Interestingly, our results showed that inhibition of RNA polymerase II C-terminal domain phosphorylation increased the number of transcribed mRNAs. However, some of these mRNAs were truncated and lacked polyadenylation, which resulted in decreased translation. These results suggest that phosphorylation of RNA polymerase II C-terminal domain is critical for linking transcription and posttrancriptional processing of mRNA expressed from extrachromosomal DNA.

## Introduction

Pharmacological inhibitors of cyclin dependent kinases (PCIs) represent a heterogeneous group of compounds that are defined by their capacity to inhibit preferentially cyclin dependent kinases (CDKs) involved in cell cycle regulation (CDK1, CDK2, CDK4, CDK6 and CDK7), transcription (CDK7 and CDK9), or neuronal function (CDK5) [1]. Since many CDKs are critical regulators of cellular division, the pharmaceutical industry has been focused on the discovery and development of pharmacological CDK inhibitors as potential anticancer drugs [2].

**Figure 1:**
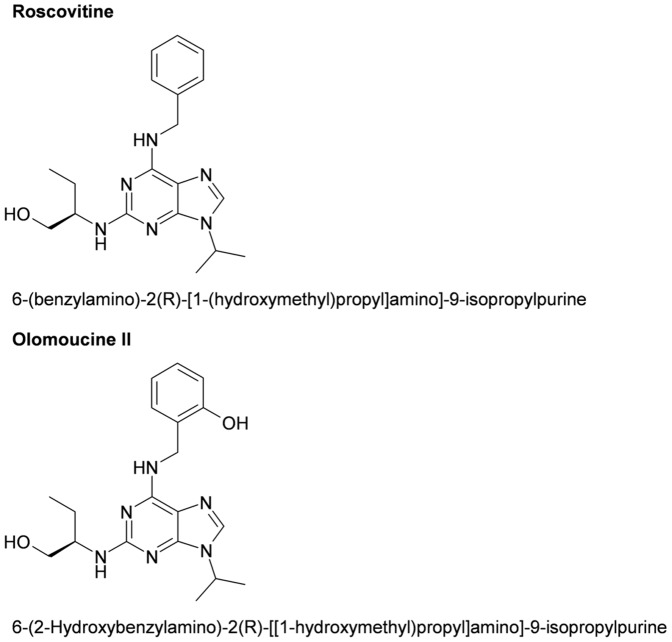
Olomoucine II differs from roscovitine only by an additional hydroxyl group on 6-benzyl group.

Olomoucine II (OCII; 6-(2-Hydroxybenzylamino)-2(R)-[[1-hydroxymethyl)propyl]amino]-9-isopropylpurine) is a 2,6,9-trisubstituted purine derivative and, like roscovitine (Rosc), is a potent reversible inhibitor of CDKs. Purine-derived CDK inhibitors preferentially target CDKs 1, 2, 5, 7 and 9, because of a shared capacity for competing for the ATP-binding pocket within CDKs and arresting the cell cycle in G2/M phase. OCII and Rosc are further known to induce the nuclear accumulation of the tumor suppressor protein p53 thereby promoting its role as a transcription factor [3], [4]. Despite the structural similarity between these two PCIs (OCII differs from Rosc only by the presence of an additional *ortho*-hydroxyl group on benzyl ring, see **Figure 1**), OCII displays about 10-fold higher inhibitory effect on CDK9, and preferentially inhibits the S to G2 transition [5].

While CDK inhibitors clearly hold great promise as anticancer drugs, interest in their capacity to inhibit viral replication keeps growing [6], [7]. Recently, CDKs have been shown to be required for the efficient replication of a number of clinically-important viruses, including papillomaviruses, human immunodeficiency virus type-1 (HIV-1), human cytomegalovirus (HCMV), herpes simplex virus (HSV) type 1 and HSV-2 [8], [9]. Rosc, olomoucine (the precursor of OCII) and several non-purine derived PCIs (such as flavopiridol, FVP) inhibit the replication of viruses that are known to affect cell cycle progression, including HCMV [6], HSV [10] and HIV-1, -2 [11].

Virus gene transcription is often dependent on host cell factors such as RNA polymerase II (RNAP II) [12]. C-terminal domain (CTD), the largest subunit of RNAP II, is an essential transcriptional enhancer that participates in organizing transcription foci within the nucleus and in pre-mRNA processing steps. Phosphorylation of a heptapeptide repeat sequence within the CTD was recognized to play an important role in the transcriptional process. CDK7 and CDK9 are subunits of general transcription factors responsible for phosphorylation of the large subunit of RNAP II and further transition from abortive to productive pre-mRNA elongation by RNAP II. A study using step by step *in vitro* transcription in mammalian nuclear extracts indicated that serine 5 (Ser 5) of RNAP II is phosphorylated first in the initiation complex by CDK7 and recruits the capping enzyme; in contrast Ser 2 is phosphorylated by CDK9 upon entry into elongation and recruits factors for co-transcriptional 3′end processing [13].

Our previous studies have determined that OCII is a more potent inhibitor of CDK activities, shows greater selectivity towards CDK9 and is a more potent antiviral drug than Rosc [5], [14].

To understand the basis for these differences in activity, we focused on their effects on RNA polymerase II phosphorylation and expression of genes regulated by viral promoters. We report that OCII inhibits expression from viral promoters at substantially lower concentrations than Rosc. Consistent with observations made with Rosc, OCII was effective only towards viral promoter not integrated into the genome of host cells. Both PCIs increased the initiation of pre-mRNA synthesis from all tested viral promoters, however only transcription from viral promoters integrated into the cellular genome led to production of full-length transcripts. Transcription from extrachromosomal viral promoter mainly generated short, abortive transcripts in PCI treated cells.

## Materials and Methods

### Cell Culture

The African green monkey kidney cell line CV-1 (expressing wild type p53), COS-1 cells (established from CV-1 by transformation with an origin-defective mutant of SV40), the human non-small cell lung carcinoma cell line H1299 (p53-null) and the human breast adenocarcinoma cell line MCF-7 (physiologically expressing low level of wild type p53) were maintained in Dulbecco’s modified Eagle medium (DMEM) containing 10% fetal bovine serum, 300 µg/ml L-glutamine, 105 IU/ml penicillin and 100 µg/ml streptomycin. All cell lines were obtained from the American Type Culture Collection.

### Generation of Stable Cell Lines

The plasmids pCEP4-Tat and pHIV-lacZ were obtained from the NIH AIDS Research & Reference Reagent Program [15], [16]. pCEP4-Tat contains HIV-1SF2 *Tat*, while pHIV-lacZ contains all of the U3 region, part of the R region (including the TAR) and the HIV-1 3′LTR driving lacZ, and pSV-lacZ (Promega) contains lacZ under control of the SV40 early promoter. DNA transfections were carried out using Lipofectamine 2000 (Invitrogen) according to the manufacturer’s instructions. The H1299-Tat and H1299-HIV cell lines were generated by transfection of H1299 cells with pCEP4-Tat alone, or with both pCEP4-Tat and pHIV-lacZ plasmids, respectively. Two days post-transfection, the cells were selected with 250 µg/ml hygromycin B (Merck Biosciences) to generate stable clones with plasmid DNA integrated into the host genome.

### Production of PCIs

Both roscovitine and olomoucine II have been synthesized in-house by experienced medicinal chemist. The compounds have been purified by column chromatography and re-crystallizations. The purity was confirmed by HPLC/DAD/MS and elemental analyses to be approximately 99.6%. Structures were confirmed by 1H-NMR and 13C-NMR (Bruker Avance III 400 spectrometer) and mass spectrometry (Finnigan MAT, LCQ ion trap) [3], [4].

### β-galactosidase Assay

Cells were harvested 12 h post-transfection and lysed by sonication in 0.25 M TrisHCl, pH 7.5. To measure β-galactosidase activity, 30 µl of cell lysate (50 µg of total protein) was mixed with 3 µl of buffer 1 (0.1 M MgCl_2_, 4.5 M β–mercaptoethanol), 66 µl of ONPG buffer (4 mg/ml o-nitrophenyl-β-D-galactopyranoside in 0.1 M sodium phosphate buffer, pH 7.5) and 201 µl of 0.1 M sodium phosphate buffer, pH 7.5. The reaction was incubated for 30 min at 37°C, stopped with 500 µl of 1 M Na_2_CO_3_ and the absorbance was determined at 420 nm in a microplate reader. Samples were analyzed in three independent biological replicates. The error bars illustrate the standard deviation.

### Immunoblotting

Cells were detached with cell scrapers, washed three times with ice-cold PBS and resuspended in lysis buffer (50 mM TrisHCl, pH 7.4, 250 mM NaCl, 5 mM EDTA, 50 mM NaF, 1 mM Na_3_CO_4_, 1% Nonidet P40) containing protease inhibitor cocktail and phosphatase inhibitor cocktail 2 (Sigma-Aldrich). 20 µg of total proteins were separated by SDS-polyacrylamide gel electrophoresis (SDS-PAGE) on 8% or 10% gels and transferred onto nitrocellulose membranes. Membranes were blocked in 5% milk and 0.1% Tween 20 in PBS and probed overnight with specific monoclonal antibodies or rabbit polyclonal sera. Primary antibodies specific for RNA polymerase II CTD (all from Santa Cruz Biotechnology) included: (i) N-20 used at 1 µg/ml; (ii) H14, specific for form phosphorylated at Ser 5, used at 6 µg/ml; (iii) H5, specific for the form phosphorylated at Ser 2 used at 4 µg/ml. Additional primary monoclonal antibodies used in immunoblots included (iv) anti-p53 (DO-1, in house [17]), used at 1 µg/ml; (v) anti-p21^WAF−1^ (118, in house [18]), used at 1 µg/ml; (vi) anti-actin (A-2066, Sigma-Aldrich) used at 1 µg/ml; (vii) anti-T-antigen (419, in house [19]), used at 1 µg/ml. All primary antibodies were diluted in PBS containing 5% milk and 0.1% Tween 20. Peroxidase conjugated rabbit anti-mouse immunoglobulin or porcine anti-rabbit immunoglobulin antisera (DAKO Glostrup) were used as the secondary antibodies and visualized with ECL reagents (Amersham-Pharmacia). The selected bands from immunoblots were quantified using TotalLab Quant software. Band intensities were normalized to actin as loading control, displayed values represent fold changes relative to untreated controls.

### Infection

The cell lines MCF-7 and T47D were infected with SV40 virus (MOI = 10) for 2 h in serum-free DMEM. After infection, DMEM was supplemented with 10% fetal bovine serum and either 8 µM OCII or 20 µM Rosc added immediately or 16 h post-infection. Infected cells were harvested at 45 h.

### Real-time PCR

Total cellular RNA was extracted using the RNeasy Mini kit (Qiagen) according to the protocol for cultured cells. cDNA synthesis was carried out with 500 ng of total RNA using M-MLV reverse transcriptase (Fermentas Life Sciences) in a total volume of 34 µl according to the manufacturer’s protocol. Samples in triplicate were subjected to qRT-PCR analysis using SYBR Green in a 7900 HT Fast Real-Time PCR System (all by Applied Biosystems,) at conditions: 50°C for 2 min, 95°C, 10 min, and 40 cycles of 95°C for 15 sec, 60°C for 1 min. The primer pair specific for the N-terminus of β-galactosidase cDNA were B-GAL mRNA F1 5′CTGGCGTAATAGCGAAGAGG3′ and B-GAL mRNA R1 5′AGTTTGAGGGGACGACGACAG 3′. Primers for the C-terminus of β-galactosidase cDNA were B-GAL mRNA F2 5′ TGGCGATTACCGTTGATGTTGAAG3′ and B-GAL mRNA R2 5′ CAGTAAGGCGGTCGGGATAGTTTT3′ oligonucleotides. The relative quantification of gene expression was determined by the comparative C_T_ method using *ACTB* (human beta-actin) mRNA as endogenous control, amplified with primer pair ACTB 1350F 5′GGAACGGTGAAGGTGACAGC3′ and ACTB 1561R 5′ACCTCCCCTGTGTGGACTTG3′. Results from three independent experiments were averaged. Asterisk denotes significant difference with p<0.05, determined by one-way ANOVA, The error bars illustrate the standard deviation.

## Results

### Inhibition of Gene Expression from SV40 Promoter by OCII and Rosc

Simian virus 40 (SV40) is a DNA virus that potentially causes tumors. The cellular-encoded RNA polymerase II acts to promote SV40 early gene expression and the resulting mRNA is translated into the small and large T antigens. SV40 large T-antigen exerts pleiotropic effects and was shown to be responsible for multiple functions involving regulation of virus infection and promoting tumorigenesis. Among the wide range of characterized interactions, T-antigen forms a stable complex with p53 and by promoting pRb dephosphorylation it inactivates members of the pRb family [20]–[22]. The inactivation of p53 is critical for SV40-mediated cell transformation, whereas the concomitant destabilization of the inhibitory complex between dephosphorylated pRb and the E2F family of transcription factors facilitates the transcription of target genes to promote cell cycle entry [23], [24].

**Figure 2:**
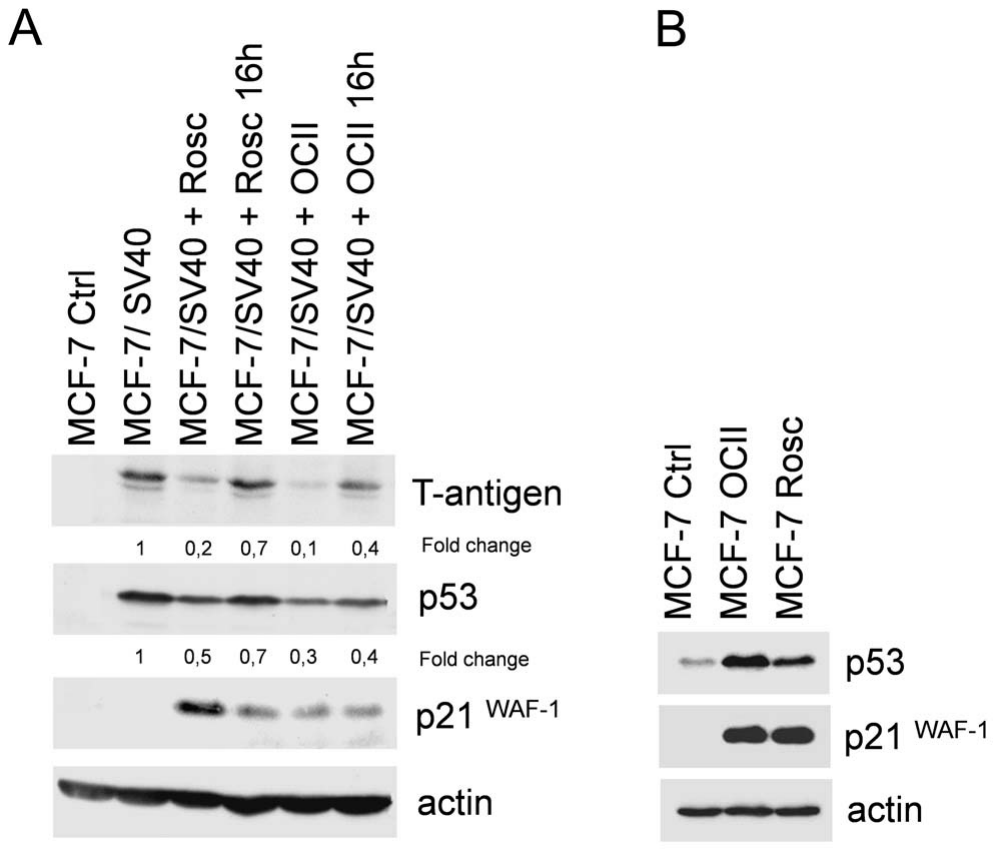
OCII and Rosc suppress T-antigen expression. (A) OCII and Rosc suppress T-antigen expression and restore p53–mediated transactivation of p21^WAF−1^ during SV40 infection. MCF-7 (wild type p53) cell line infected with SV40 was treated either with 8 µM OCII or 20 µM Rosc, either immediately or 16 h post-infection. (OCII 16 h, Rosc 16 h). Infected cells were harvested at 45 h and then analyzed for the expression of large T-antigen, p53, p21^WAF−1^ and actin by immunoblotting. (B) Inhibition of expression from viral promoter integrated into cellular genome. Cell lines CV-1 and COS-1 were treated either with 8 µM OCII or 20 µM Rosc and analyzed for the expression of large T-antigen, p53, p21^WAF−1^ and actin by immunoblotting. CV-1 cell line was used as SV40-negative control cell line (no large T-antigen expression). The numbers represent the fold changes of samples to untreated controls.

We evaluated the effect of PCIs on large T-antigen expression and its impact on p53 level and activity. The functional activity of p53 was determined by analysis of its target protein p21^WAF−1^. MCF-7 (wild type p53) cell line was infected with SV40 and the effect of OCII and Rosc on the expression of T-antigen, p53 and p21^WAF−1^ was examined. The expression of T-antigen was detected in cells infected with SV40 (**Figure 2A**). As expected, interaction of p53 with large T-antigen resulted in stabilization of wild type p53 (high level of p53 that is not degraded) and inactivation of p53 (protein p21^WAF−1^ is not transactivated) in MCF-7 cells infected by SV40. Treatment of SV40 infected cells with OCII or Rosc led to a reduced level of large T-antigen, an observation consistent with a growing body of evidence that CDKs are required for efficient expression from SV40 early genes [25]. T-antigen production was more markedly reduced when OCII and Rosc were added immediately to the MCF-7 infected cells rather than 16 h post-infection. These data indicate that the effect of PCIs is more efficient at earlier time points post-infection. In line with these findings, we also observed inhibition of p53 accumulation in MCF-7 cells and recovery of its function, represented by transactivation of the p53 target protein, p21^WAF−1^ (**Figure 2A**).

To distinguish the effect of each PCI on p53 and p21^WAF−1^ protein levels and activity, non-transfected MCF-7 cells were treated with OCII or Rosc. Both agents enhanced the protein levels of p53 and p21^WAF−1^ (that is tightly controlled by p53) in MCF-7 cells that normally leads to cell cycle arrest (**Figure 2B**).

**Figure 3:**
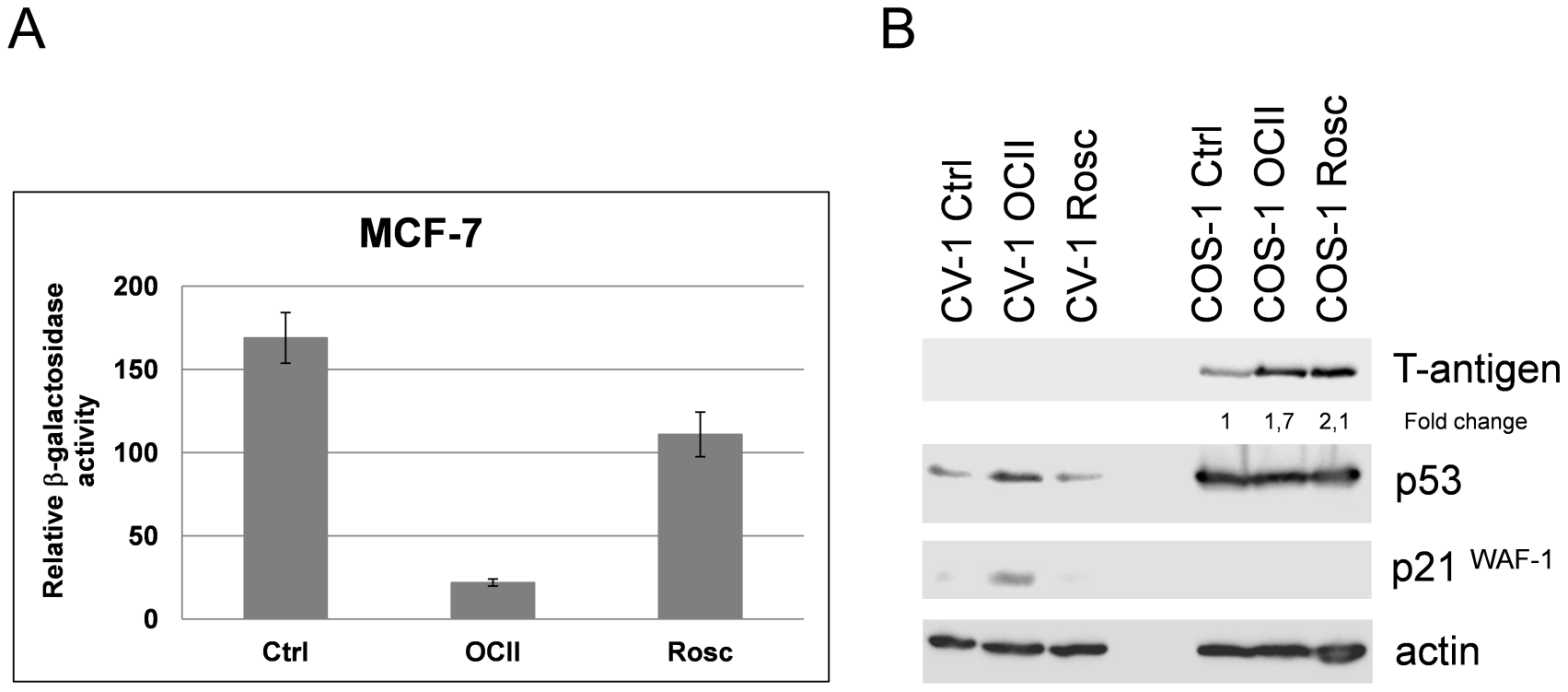
OCII and Rosc suppress expression from extrachromosomal genetic elements. (A) MCF-7 cells transfected with pSV-lacZ were treated either with OCII (8 µM) or Rosc (20 µM). Relative levels of β-galactosidase activity were determined in a chromogenic (ONPG) enzyme assay. The Y axis represent**s** relative β-galactosidase activity and the error bars illustrate the standard deviation of three independent biological replicates. (B) OCII and Rosc induce wild type p53-mediated transactivation of p21^WAF−1^. MCF-7 (wild type p53) cell line was treated either with OCII (8 µM) or Rosc (20 µM) for 24 h and analyzed for the expression of p53, p21^WAF−1^ and actin by immunoblotting. The numbers represent the fold changes of samples to untreated controls.

To ascertain whether PCIs inhibited only large T-antigen expression or whether their effects were connected generally with SV-40 promoter transcription, we tested their inhibitory effects on an isolated viral promoter within a plasmid vector. MCF-7 cells transiently transfected with plasmid pSV-lacZ were treated with OCII or Rosc and relative β-galactosidase activities determined. We observed significant (p>0.05) reductions of β-galactosidase activity in cells treated with PCIs (**Figure 3A**) indicating inhibition of β-galactosidase expression from the viral SV-40 promoter. These results showed that PCIs affected gene expression from the SV40 promoter.

### The Effect of PCIs on Expression of T-antigen Integrated Into the Cellular Genome

It has been described that virus replication or transcription can be more sensitive to CDK9 or p-TEFb activity than transcription of cellular genes [26], [27]. Moreover, while we observed inhibition of expression from viral promoter, expression of cellular genes *TP53* and *CDKN1A* increased (**Figure 2B**). This observation led us to test the effect of OCII and Rosc on T-antigen expression in COS-1 cells, where the sequences encoding large T-antigen are integrated into the cellular genome. COS-1 is an African green monkey kidney cell line that was established from CV-1 by transformation with an origin-defective mutant of SV40. CV-1 cell line was used as a control. While expression of T-antigen in SV40-infected cells (SV40 genome was not integrated) was inhibited by PCIs (**Figure 2A**), in COS-1 cells OCII and Rosc treatment stimulated large T-antigen expression, while p53 levels remained constant and the protein transcriptionally inactive (p21^WAF−1^ was not activated). Conversely, in the control CV-1 cells, the transcriptional activity of p53 and in turn p21^WAF−1^ levels increased after the PCI treatment (**Figure 3B**). These results indicate that PCIs may have either an inhibiting or a stimulating effect on a viral promoter, depending on its location.

### Inhibition of Expression from the HIV Promoter

To further analyze the effect that PCIs exert on expression from integrated and extrachromosomal viral DNA elements, we decided to test expression from HIV promoter, taking advantage of the fact that transcription from the HIV genome is sensitive to CDK9 activity and can be suppressed by PCI treatment at concentrations that do not impair cellular transcription [26], [27]. HIV requires a viral transactivator called Tat (Trans-Activator of Transcription) to stimulate the processivity of RNAPII and initiation from the viral LTR promoter. In the absence of Tat, only short, abortive transcripts are generated.

Transcription elongation factor p-TEFb represents a cellular co-factor for HIV-1 Tat transcriptional activation and it was found that CDK9 interacts functionally with the transactivation domain of Tat, illustrated by the fact that CDK9 depletion blocks Tat-dependent transactivation and activity [28], [29]. Tat is also phosphorylated by CDK2 and this modification is important both for HIV-1 transcription and activation of integrated HIV-1 provirus [30]. It was described that Roscovitine as CDK2 and CDK9 inhibitor effectively suppress wild type and resistant HIV-1 mutants and could selectively sensitize HIV-1-infected cells to apoptosis at concentrations that did not impede the growth and proliferation of uninfected cells [31]–[33].

**Figure 4:**
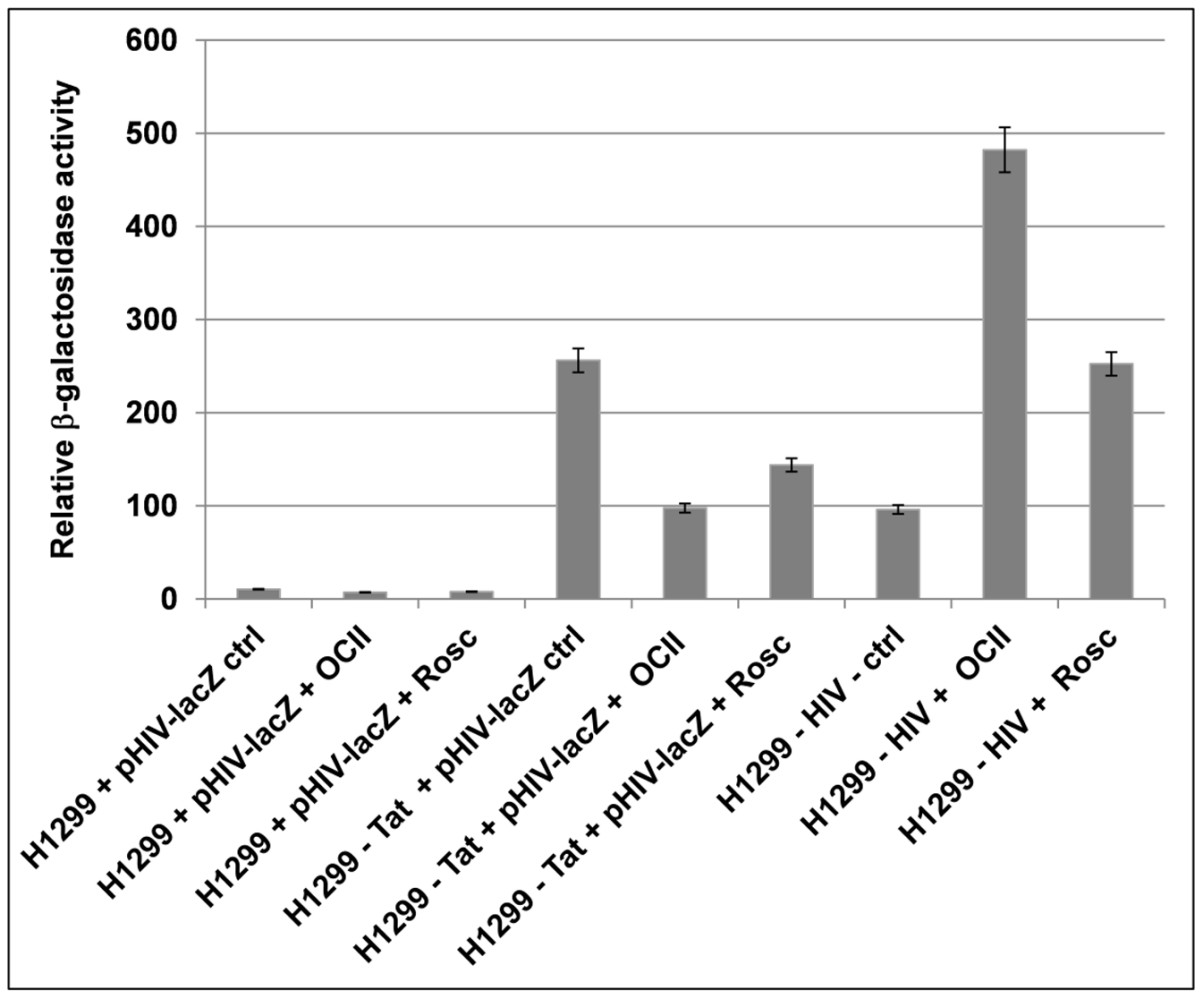
Inhibition of expression from extrachromosomal HIV promoter by PCI. A β-galactosidase reporter assay was used to measure expression from the promoter within the pHIV-lacZ plasmid following transfection into a Tat-expressing cell line (H1299-Tat). Results were compared with expression from Tat-expressing cell line with pHIV-lacZ stably integrated into the cellular genome (H1299-HIV). The requirement for Tat to promote expression from HIV promoter was illustrated in H1299 (Tat-negative) control cells transiently transfected with pHIV-lacZ. The Y axis represents relative β-galactosidase activity and the error bars illustrate the standard deviation of three independent biological replicates.

We constructed two stable cell lines: H1299-Tat (with incorporated plasmid pCEP4-Tat) and H1299-HIV (with incorporated plasmids pCEP4-Tat and pHIV-lacZ). Control cell line H1299 and H1299-Tat cells were transiently transfected with a plasmid encoding β-galactosidase under the control of the promoter in the HIV LTR (pHIV-lacZ) and together with H1299-HIV cells were treated with OCII or Rosc followed by β-galactosidase activity measurement. The expression of the reporter in H1299-Tat stable cell line transiently transfected with pHIV-lacZ was inhibited by PCI treatment and more efficiently by OCII than by Rosc (**Figure 4**). However, an inverse effect of PCI treatment on the HIV promoter integrated into the cellular genome (H1299-HIV cells) was observed and the expression of β-galactosidase in H1299-HIV cells increased about 4-fold following treatment with OCII or 2.5-fold following treatment with Rosc (p<0.05) (**Figure 4**). These findings are in agreement with results obtained with SV40 (**Figure 3B**) and indicate that effect of PCIs is not limited to one type of virus promoter, and is dependent on virus promoter extrachromosomal localization.

### Phosphorylation of the C-terminal Domain of RNA Polymerase II

**Figure 5:**
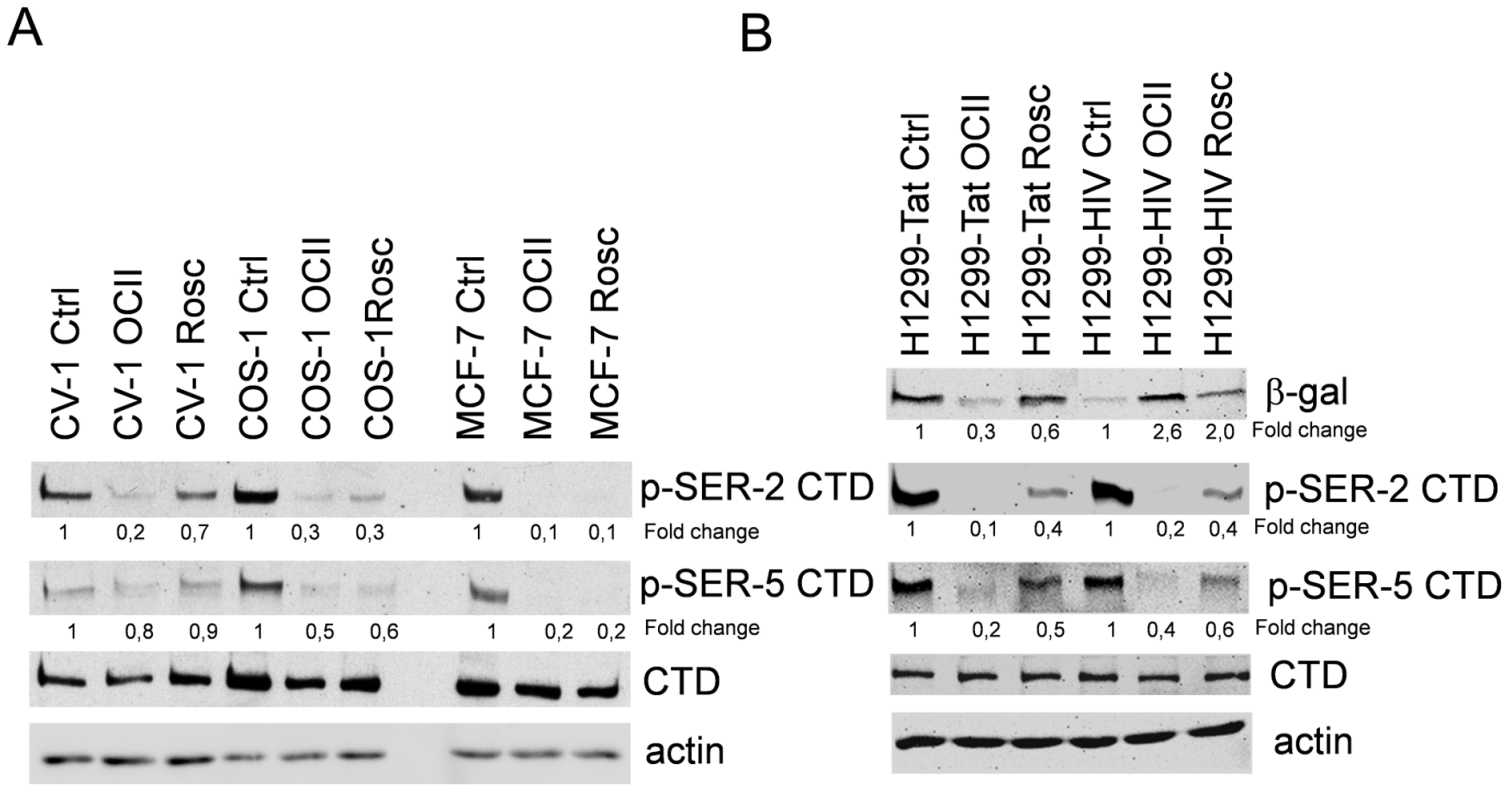
The effect of OCII and Rosc on phosphorylation of RNA polymerase II CTD. (A) Phosphorylation of RNA polymerase II CTD in wild type p53 cells either with stably integrated SV40 (COS-1) or SV40-negative cells (CV-1) was determined. The phosphorylation of both Ser 2 and Ser 5 was inhibited after 12 h treatment either with 8 µM OCII or 20 µM Rosc in all tested cell lines. (B) Comparison of RNA polymerase II CTD phosphorylation in cells expressing Tat with pHIV-lacZ stably integrated into cellular genome (H1299-HIV) or with pHIV-lacZ as a part of extrachromosomal DNA (H1299-Tat). The phosphorylation of Ser 2 and Ser 5 in both cell lines was inhibited after PCI treatment for 12 h. The numbers represent the fold changes of samples to untreated controls.

Modulation of expression from the HIV promoter by PCI treatment may be mediated by transcriptional regulation. The C-terminus of the largest subunit of RNAP II contains a key regulatory domain referred to as CTD (C-terminal domain). Therefore, we investigated the effect of PCIs on phosphorylation of CTD at positions Ser 2 and Ser 5. The levels of both Ser 2 and Ser 5 phosphorylation in OCII or Rosc treated COS-1, CV-1 and MCF-7 cells were substantially reduced (**Figure 5A**). Although the phosphorylation of RNAP II was suppressed by PCIs, the expression from viral promoter integrated into the host cell genome in H1299-HIV cells was enhanced (**Figure 5B**). This result indicated that CTD phosphorylation was essential only for the expression from the extrachromosomal virus promoter.

### Rate of RNA Synthesis and RNA Integrity with Respect to Gene Localization

To study the effect of PCIs on β-galactosidase expression (**Figure 4** **and** **5B**) and its correlation with transcription, we determined β-galactosidase mRNA levels using qRT-PCR in stable cell lines H1299-HIV and H1299-Tat transiently transfected by pHIV-lacZ.

Previous work described the importance of CTD phosphorylation for RNA posttranscriptional processing rather than for RNA synthesis, where Serine-5 phosphorylation was associated with recruitment of capping enzymes to 5′ ends, whereas Ser 2 phosphorylation was associated with recruitment of cleavage and polyadenylation factors to the 3′ ends [34].

**Figure 6:**
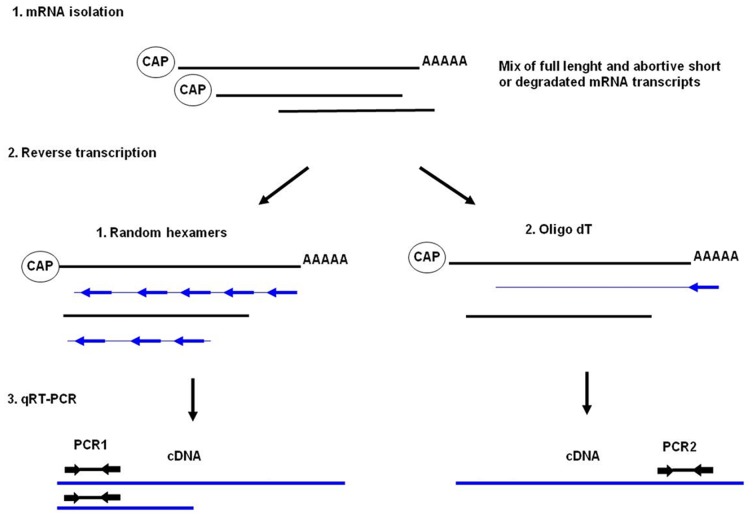
The experimental setup: 1. Total RNA was extracted. 2. Reverse transcription was performed in two different setups i) using random hexamers to collect all possible types of RNA transcripts and ii) using oligo dT primers to gain polyadenylated types of RNA transcripts. 3) Real-time PCR with primers designed to specifically recognize N- and C-terminus of β-galactosidase cDNA was used to amplify sequences at both 5′-end (PCR-1) and 3′-end (PCR-2) of β-galactosidase RNA transcripts.

Therefore, we developed real time PCR based assays to analyze the amount of RNAs and its polyadenylation. Random hexamers were used to quantify total transcribed β-galactosidase whereas oligo dT primers were used to analyze only polyadenylated RNA. The integrity of 5′ or 3′ end of RNA was assessed using PCR primer pairs amplifying the corresponding parts of the RNA termini (**Figure 6**).

**Figure 7:**
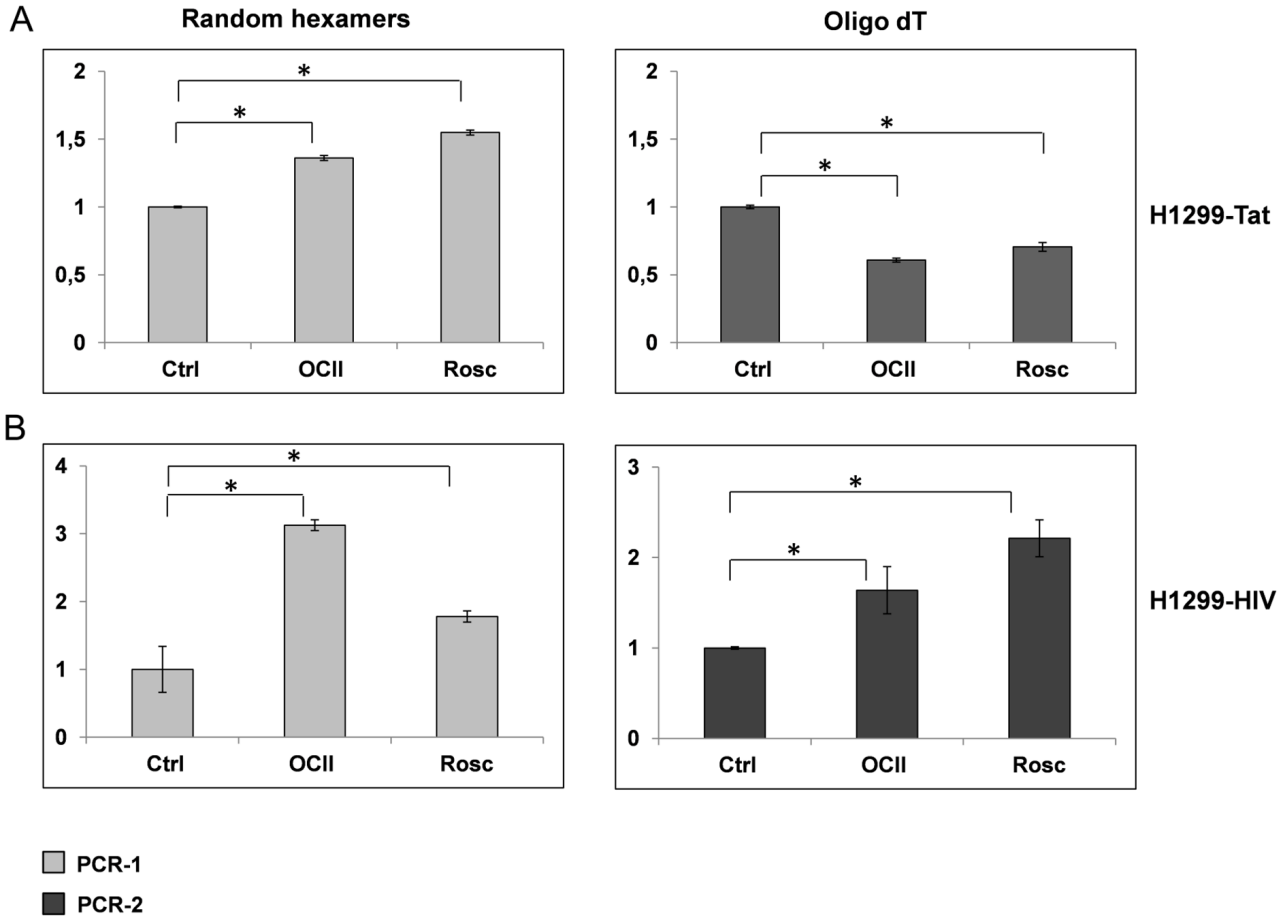
The effect of PCIs on the integrity of synthesized RNA. We compared amounts of total and full length polyadenylated transcripts of β-galactosidase gene after treatments with OLII and Rosc. We quantified total and full length transcripts of β-galactosidase gene in cell lines H1299-HIV (B) and H1299-Tat transiently transfected by pHIV-LacZ (A) by using qRT-PCR. PCIs stimulated the RNA synthesis from viral promoters ((B) H1299-HIV PCR-1, Random hexamers (A) H1299-Tat, PCR-1, Random hexamers) however viral promoters incorporated in the cellular genome generated higher amounts of full length RNA transcripts ((B) H1299-HIV, PCR-2, OLIGO dT). The amount of full length polyadenylated transcripts produced from extrachomosomal viral promoters after treatments with PCIs were significantly lower ((A) H1299-Tat, PCR-2, OLIGO dT). Data were determined by relative quantification with control PCR-1 and control PCR-2 as the calibrators. The Y axes represent the fold change of β-galactosidase RNA transcripts after PCIs treatment to the amount of β-galactosidase RNA transcripts in controls. The error bars illustrate the standard deviation of three independent biological replicates.

We found that PCIs stimulated the initiation of RNA synthesis from viral promoters regardless of their localization, as suggested by the increased levels of PCR-1 transcripts after PCI treatments compared to control PCR-1 (p<0.001) (**Figure 7A** **,** **Figure 7B** random hexamers). However, full length polyadenylated RNA transcripts were generated to a large extent only from viral promoters incorporated in the cellular genome (increased levels of PCR-2 after treatments compared to control, (p<0.001), **Figure 7A**, Oligo dT). Conversely, transcripts produced from extrachromosomal viral promoters after PCI treatments were probably much shorter and abortive because of reduced amounts of amplified PCR-2 products from cDNA templates generated using oligo dT primers in H1299-Tat cells (**Figure 7B**, Oligo dT). We also tested the levels of Tat mRNA after OCII and Rosc treatments and did not find any significant changes (**Figure S1**).

### Inhibition of Expression from the HIV Promoter by Flavopiridol

Inhibitors of CDK9 have been characterized as inhibitors of viral transcription and replication [2]. We tested whether the effect on extrachromosomal and integrated viral promoter is uniform for these agents. Flavopiridol (FVP) is a highly specific P-TEFb (CycT1:CDK9) kinase inhibitor that dramatically reduces the global levels of Ser 2 phosphorylated CTD [27]. We therefore tested the influence of FVP on RNAP II CTD phosphorylation, RNA synthesis and protein expression from viral promoters (**Figures S2, S3**). H1299-Tat and H1299-HIV cells were treated with two concentrations of FVP (25 and 100 nM). Both doses decreased the expression of mRNA from extrachromosomal HIV promoter in H1299-Tat cells (p<0.05) and in turn led to a reduction of β-galactosidase protein. The lower concentration of FVP (25 nM) did not change the expression of β-galactosidase from HIV promoter incorporated into the genome of H1299-HIV cells (p<0.05). In contrast, 100 nM FVP induced expression from this promoter (p<0.05) and increased the level of β-galactosidase protein similarly to OCII and Rosc treatments **(Figures S2, S3**). FVP in contrast to OCII and Rosc did not increase the rate of mRNA synthesis from both viral promoters (except higher concentration of FVP in H1299-HIV cells, p<0.05). However, FVP impacted on mRNA elongation initiated from these promoters as indicated by differences in the levels of N- and C-terminal β-galactosidase PCR products.

## Discussion

We have demonstrated that OCII is a potent inhibitor of viral transcription with an inhibitory effect stronger than Rosc. Activities of both agents have been variously associated with the inhibition of CDK1/cyclin B, CDK2/cyclin E, CDK2/cyclin A and to a lesser extent CDK4/cyclin D. In addition to inhibitory effects on kinases directly involved in the regulation of the cell cycle, OCII and Rosc also inhibit CDK7/cyclin H and CDK9/cyclin T, the non-cell cycle kinases [35], [36]. OCII is more selective for CDK9 than Rosc, whereas CDK2 and CDK7 activities are affected approximately to the same extent by both drugs. On the other hand Rosc is a stronger inhibitor of CDK1, CDK4 and Erk2 [5].

We observed that OCII and Rosc mediated suppression of large T-antigen expression during SV40 infection. The reduced level of T-antigen was connected with releasing p53 from inhibitory complex with T-antigen and with restoration of p53 transcriptional activity. Interestingly, this suppression was dependent on the time period of the MCF-7 cell line treatment, as the depletion of T-antigen was significantly lower when PCIs were added 16 h post-infection with SV40. Likewise, time-dependent effects have also been described for Rosc treatment in HCMV-infected cells, where the level of inhibition was dependent on whether the drug was added before or after the onset of viral DNA replication [37]. Addition of Rosc at the time of infection altered the accumulation and processing of selected early genes and inhibited HCMV DNA replication. Delaying the addition of the drug until 6 h post-infection abrogated the deleterious effect on early gene expression and viral DNA synthesis [37], [38]. Comparable effects have also been observed in cells infected with herpes simplex virus [25]. These data indicate that the mechanism of OCII and Rosc inhibitory effects on SV40 could be the same as for HCMV and the impact of PCIs is much stronger when they are added before SV40 early gene expression and DNA replication. We confirm the influence of PCIs on expression from SV40 promoter using pSV-lacZ vector, where a stronger effect of OCII was identified. Transcription of viral genes was described to be more sensitive to inhibition of CDK9 or p-TEF-b activity than cellular genes. We tested if the inhibitory effects of OCII and Rosc on expression from viral promoters changed when the virus is incorporated into cellular genome. We utilized the COS-1 cell line containing defective mutant of SV40. We found that PCI treatments surprisingly increased the transcription of T-antigen in COS-1 cells. Likewise, the complex of p53/T-antigen remained stable and the transcriptional activity was limited. The same results were obtained with stable cell lines H1299-Tat and H1299-HIV, where the treatment with PCIs increased the expression from HIV promoter and the effect of OCII was markedly stronger.

Cassé et al. reported that low concentrations of actinomycin D and α-amatinin (inhibitor of RNA transcription and inhibitor of RNAP II) enhance transcriptional elongation from the HIV-1 LTR. This effect may result from increased CDK9 activity induced by transcriptional arrest. In HeLa cells, ∼50% of CDK9/cyclin T complexes are kinase-inactive and bound to 7SK small nuclear RNA [39]. The inhibition of transcription with actinomycin D or α-amatinin induces rapid release of active CDK9/cyclin T complexes and thereby promotes an increase in the proportion of RNAP II phosphorylated on its CTD [40]. CTD phosphorylation can also be initiated by osmotic shock, UV-irradiation, okadaic acid or heat shock. All these factors activate HIV LTR transcription, however this enhancement can be suppressed by CDK9 inhibitors [39]. OCII and Rosc induce transcription stress [5] and would therefore be expected to release active CDK9/cyclin complex. Therefore we tested the level of CTD RNAP II phosphorylation in cells treated by OCII and Rosc.

The C-terminal domain of largest subunit of RNA PII contains a series of “YSPTSPS” heptapeptide repeats that are multiply-phosphorylated during eukaryotic transcription. In the process of CTD phosphorylation at Ser 2 by P-TEFb, the CDK9/cyclin T1 complex has been implicated in productive elongation and the 3′end processing of the primary transcripts such as 3′end cleavage and polyadenylation [41], [42]. CTD phosphorylation at Ser 5 by TFIIH kinase is a critical step in mRNA synthesis, namely RNAP II promoter clearance (for transition from initiation to early elongation) and mRNA 5′-capping. We found that OCII and also Rosc dramatically decreased the phosphorylation of CTD, especially at Ser 2 but also at Ser 5 in all tested cell lines. Hong et al. [43] demonstrated that inhibition of TFIIH resulted in defective mRNA capping for the majority of yeast genes. This led to the degradation of mRNAs by exonucleases, resulting in a dramatic reduction in steady-state mRNA levels. However, inhibition of the TFIIH kinase did not significantly affect other transcriptional processes, such as overall RNAP II density and CTD phosphorylation at Ser 2. CDK9, the main CTD Ser 2 kinase, is critical for 3′end processing of pre-mRNA, as well as for RNAP II transcription but to a lesser extent [13], [44]. OCII and Rosc as potent inhibitors of CDK9 and CDK7 could reduce the expression from viral promoters in both ways. We demonstrate that PCIs increase the initiation of RNA synthesis from viral promoters, but newly produced transcripts are immediately degraded due to defects in pre-mRNA elongation, 3′end processing and possibly in 5′-capping as well. Importantly, we observed this effect significantly in case of extrachromosomal plasmids and viral infections but only if the viral promoter was not incorporated into the cellular genome. The above mentioned variable effects of OCII and Rosc on expression from viral promoters are undoubtedly connected with localization of these promoters in host chromosome. Pirngruber et al. described that CDK9 plays an important role in the regulation of transcription not only by directing RNAP II activity but also through the modification of chromatin binding and modifying factors. CDK9 guides a complex network of chromatin modifications including histone H2B monoubiquitination (H2Bub1), H3 lysine 4 trimethylation (H3K4me3) and H3K36me3. CDK9 directs H2Bub1, which influences the accessibility of the newly synthesized mRNA or the recruitment of 3′end processing machinery by modifying the chromatin structure [45]. CDK9 knockdown increases the production of polyadenylated transcripts of replication-dependent histone mRNAs. Thus, CDK9 acts to integrate phosphorylation during transcription with chromatin modifications to control co-transcriptional histone pre-mRNA processing [45]. The increase in the polyadenylation of pre-mRNA is likely to be specific for this class of mRNAs and not a general defect in polyadenylation site selection [46]. It is likely that chromatin arrangement of viral DNA is changed after incorporation in host chromosome, so the described phenomenon could help to explain the different effects of PCIs on extra- and intrachromosomal viral promoters. Our statement is in line with published results, where Zhou et al. [47] demonstrated that the presence of H3K4me3 and H3K36me3 on the HIV-1 gene decreased following CDK9 inhibition by flavopiridol. The dependence of HIV-1 transcription on chromatin modification and histone acetylation was also demonstrated by van Lint et al. [48]. They demonstrated that global hyperacetylation of cellular histones in cells latently infected with HIV-1 increases the transcriptional activation of the HIV-1 promoter. Moreover Keskin et al. [49] described the upregulation of some Primary Response Genes (PRGs) after treatment with FVP. PRGs have constitutively open chromatin architecture and show high level of preexisting H3K4me3 across the promoter region as well as H3K36me3 in coding region [50].

In summary, we show that PCIs inhibit gene expression form viral promoters by generating defects in pre-mRNA elongation and 3′end processing. The results indicate that the inhibitory effect of PCIs on transcription from viral promoters depends on their localization in cells and the CTD phosphorylation is critical for linking transcription and posttrancriptional processing of mRNA expressed particularly from extrachromosomal DNA.

## Supporting Information

Figure S1 **The expression of Tat gene in cell lines H1299- Tat and H1299-HIV.** (A) We evaluated Tat mRNA basal level in cell lines H1299-HIV and H1299-Tat. Samples in triplicate were subjected to qRT-PCR analysis using SYBR Green with the primer pair specific for Tat cDNA: TAT-F 5′ATGGAGCCAGTAGATCCTAA′3 and TAT-R 5′GGGTTGCTTTGATAGAGAAGC′3. The relative quantification of gene expression was determined by the comparative CT method using ACTB (human beta-actin) mRNA as endogenous control. The results from three independent experiments were averaged and the error bars illustrate the standard deviation. We found that in both cell lines are the comparable amounts of Tat mRNAs (A) that did not shown any significant changes after OCII and Rosc treatments ((B) - H1299-Tat, (C) – H1299-HIV). The Y axes represent the fold change of Tat RNA transcripts in H1299-Tat and H1299-HIV cell lines compared to negative control H1299 (without transfected vector pCEP4-Tat) (A) and the fold change of Tat RNA transcripts after PCI treatment to the amount of Tat RNA transcripts in controls (B, C). The error bars illustrate the standard deviation of three independent biological replicates.
(TIF)

Figure S2 **Inhibition of expression from the HIV promoter using Flavopiridol.** H1299-Tat and H1299-HIV cell lines were treated with Flavopiridol (25 nM and 100 nM) for 12 h and the levels of RNA polymerase II CTD phosphorylation on Ser-2 and Ser-5, β-galactosidase protein and actin were analyzed by immunoblotting. FVP moderately decreased phosphorylation of Ser 2 RNA polymerase II CTD and significantly decreased the level of β-galactosidase protein in H1299-Tat cells. The impact of FVP in H1299-HIV cells was dependent on its concentration. The effect of 25 nM FVP was similar in both cell lines. In contrast, 100 nM FVP (similar to OCII and Rosc) increased the level of β-galactosidase protein in H1299-HIV cells.
(TIF)

Figure S3 **The effect of Flavopiridol on the integrity of synthesized RNA.** qRT-PCR was performed in H1299-HIV and H1299-Tat cell lines treated with 25 nM and 100 nM FVP. Total RNA was extracted and reverse transcription was performed in two different setups i) using random hexamers and ii) oligo dT primers to gain all possible types of RNA transcripts. Real-time PCR with primers designed to specifically recognize N- and C-terminus of β-galactosidase cDNA was used to amplify sequences at both 5′- and 3′-end of β-galactosidase RNA transcripts. We compared the amounts of full length and short abortive transcripts of β-galactosidase gene. The effect of FVP was dependent on the concentration. 25 nM FVP did not increase expression from either viral promoter (PCR-1 random hexamers) and decreased the quantity of β-galactosidase full length mRNA transcripts (PCR-2 oligo dT). Treatment by 100 nM FVP increased the expression from HIV-promoter (PCR-1 random hexamers) and the number of β-galactosidase full length mRNA transcripts in H1299-HIV cells (PCR-2 oligo dT).
(TIF)
