# Supplementary figures and images for: Inhibition of Post-Transcriptional RNA Processing by CDK Inhibitors and Its Implication in Anti-Viral Therapy
Source: PLoS One. 2014 Feb 21;9(2):e89228. doi: 10.1371/journal.pone.0089228 (PMC3931720; doi:10.1371/journal.pone.0089228)

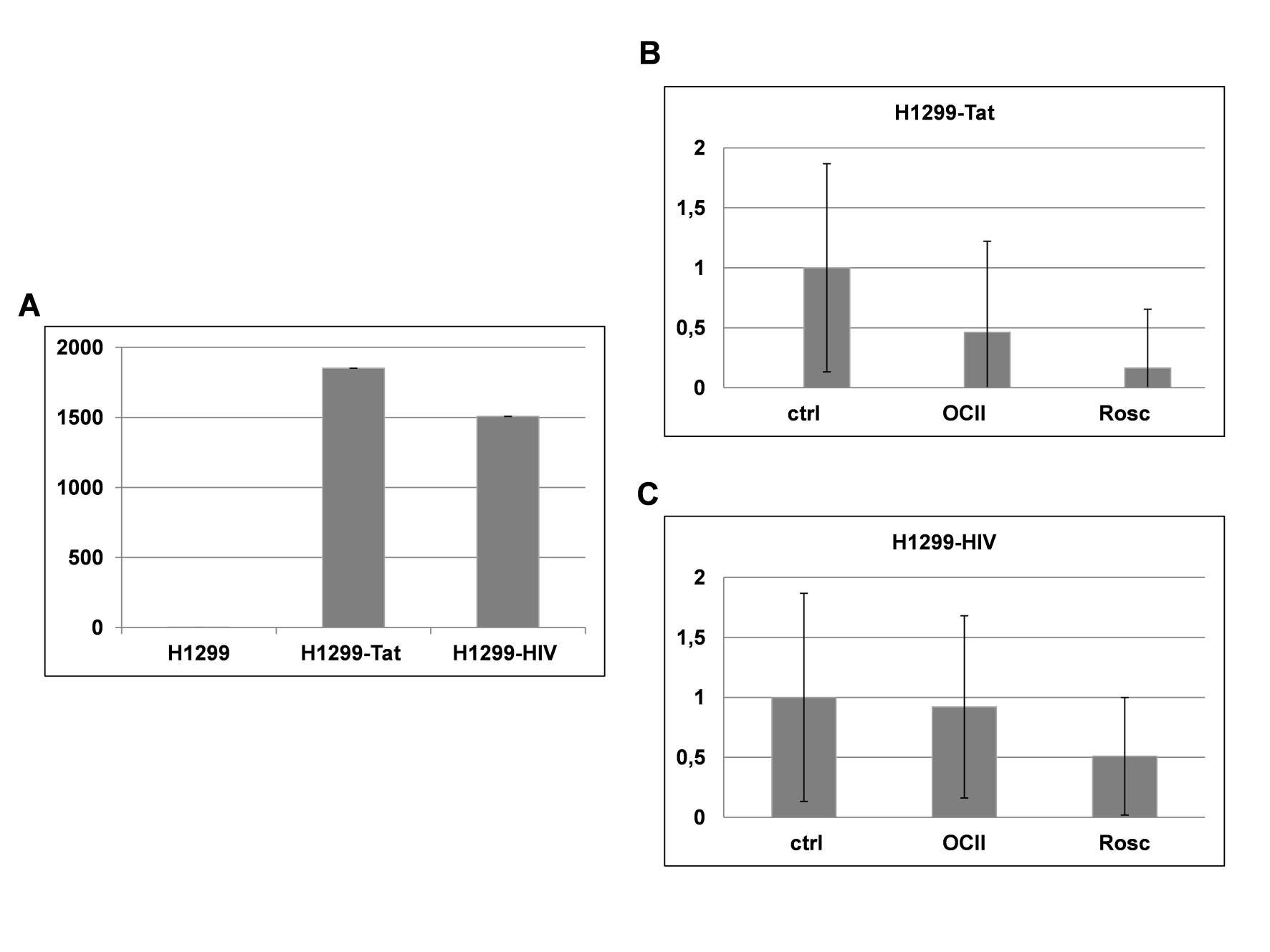

Supplement: Figure S1 — The expression of Tat gene in cell lines H1299- Tat and H1299-HIV. (A) We evaluated Tat mRNA basal level in cell lines H1299-HIV and H1299-Tat. Samples in triplicate were subjected to qRT-PCR analysis using SYBR Green with the primer pair specific for Tat cDNA: TAT-F 5′ATGGAGCCAGTAGATCCTAA′3 and TAT-R 5′GGGTTGCTTTGATAGAGAAGC′3. The relative quantification of gene expression was determined by the comparative CT method using ACTB (human beta-actin) mRNA as endogenous control. The results from three independent experiments were averaged and the error bars illustrate the standard deviation. We found that in both cell lines are the comparable amounts of Tat mRNAs (A) that did not shown any significant changes after OCII and Rosc treatments ((B) - H1299-Tat, (C) – H1299-HIV). The Y axes represent the fold change of Tat RNA transcripts in H1299-Tat and H1299-HIV cell lines compared to negative control H1299 (without transfected vector pCEP4-Tat) (A) and the fold change of Tat RNA transcripts after PCI treatment to the amount of Tat RNA transcripts in controls (B, C). The error bars illustrate the standard deviation of three independent biological replicates. (TIF) [file pone.0089228.s001.tif]

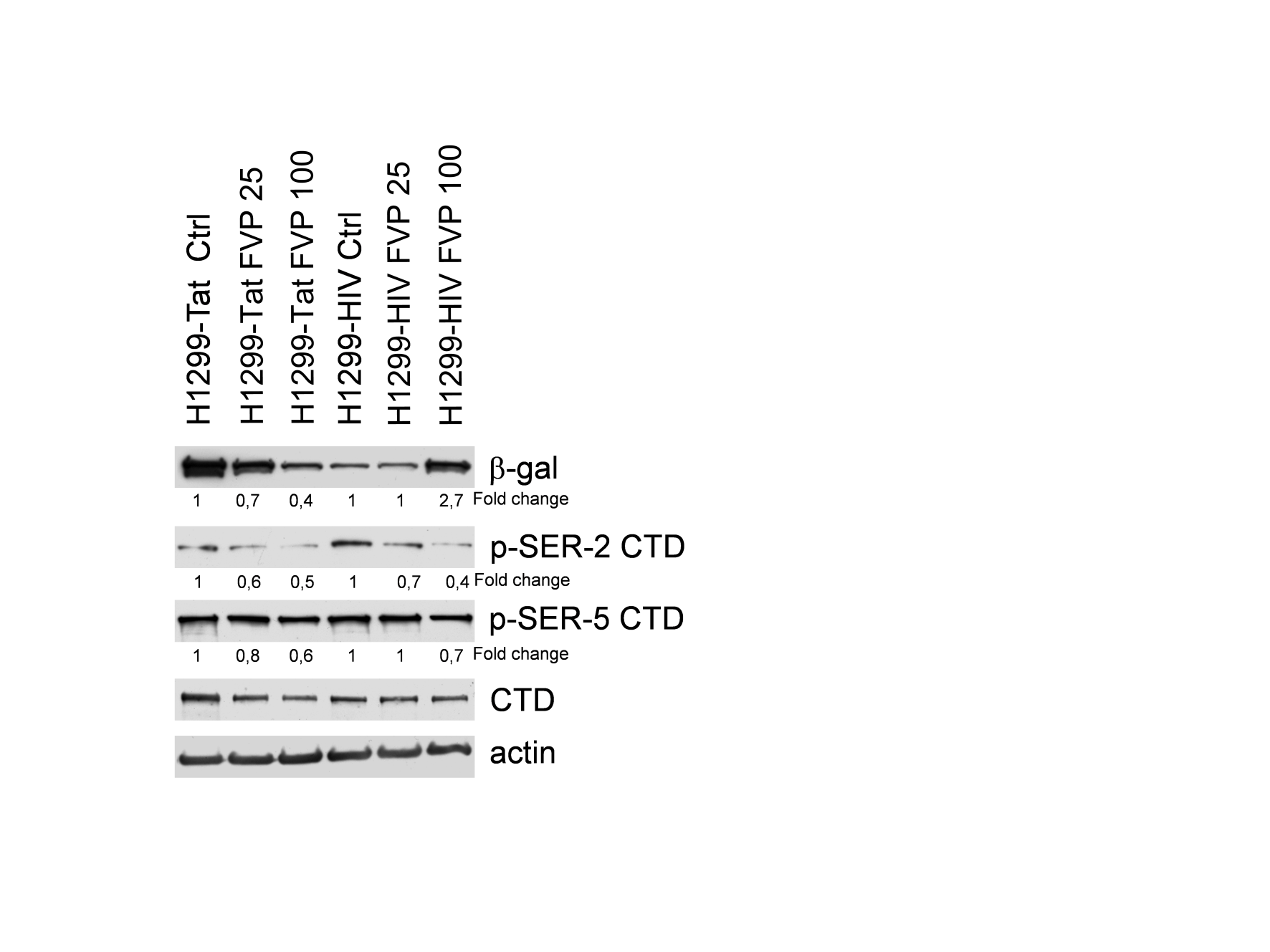

Supplement: Figure S2 — Inhibition of expression from the HIV promoter using Flavopiridol. H1299-Tat and H1299-HIV cell lines were treated with Flavopiridol (25 nM and 100 nM) for 12 h and the levels of RNA polymerase II CTD phosphorylation on Ser-2 and Ser-5, β-galactosidase protein and actin were analyzed by immunoblotting. FVP moderately decreased phosphorylation of Ser 2 RNA polymerase II CTD and significantly decreased the level of β-galactosidase protein in H1299-Tat cells. The impact of FVP in H1299-HIV cells was dependent on its concentration. The effect of 25 nM FVP was similar in both cell lines. In contrast, 100 nM FVP (similar to OCII and Rosc) increased the level of β-galactosidase protein in H1299-HIV cells. (TIF) [file pone.0089228.s002.tif]

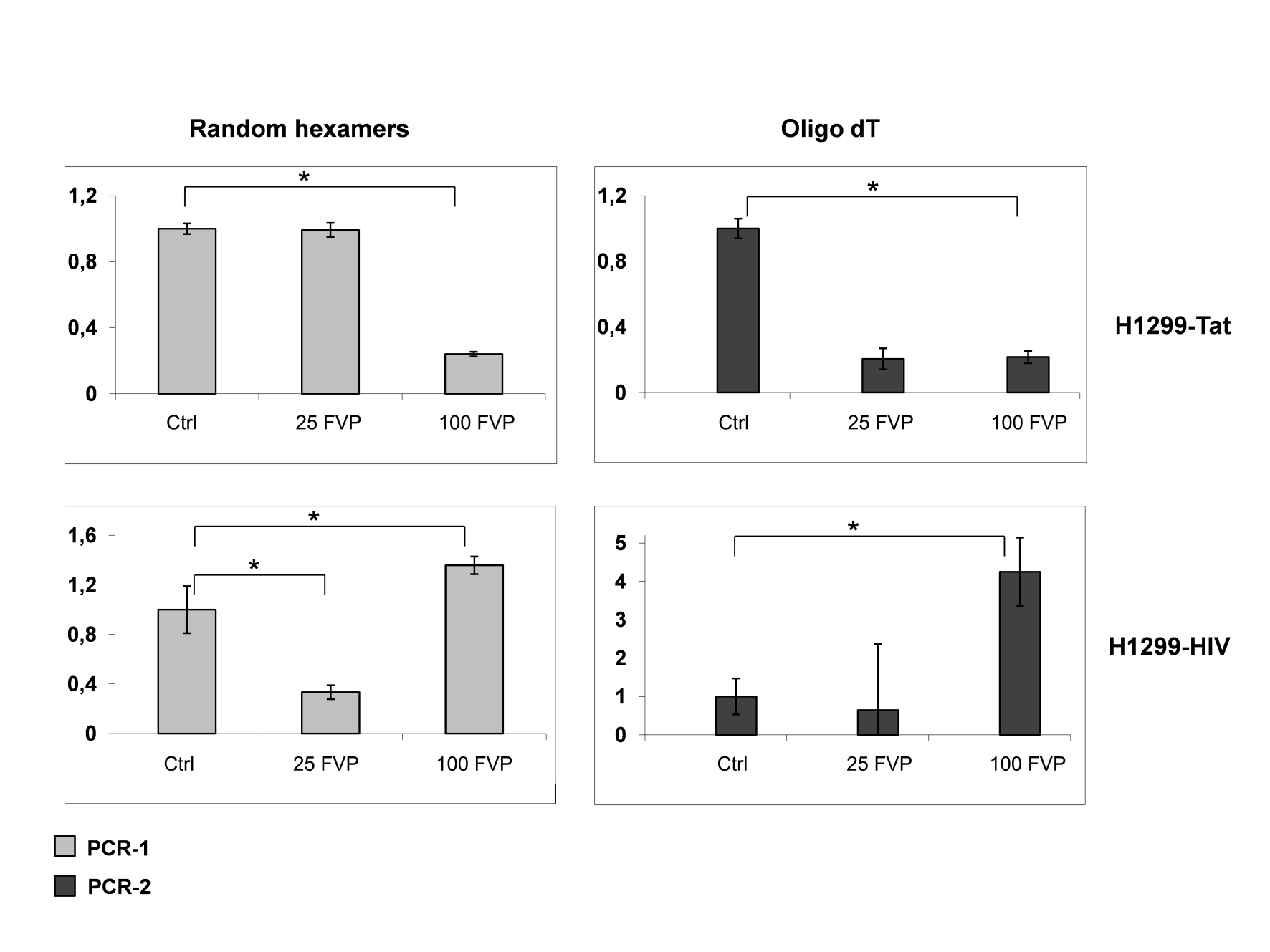

Supplement: Figure S3 — The effect of Flavopiridol on the integrity of synthesized RNA. qRT-PCR was performed in H1299-HIV and H1299-Tat cell lines treated with 25 nM and 100 nM FVP. Total RNA was extracted and reverse transcription was performed in two different setups i) using random hexamers and ii) oligo dT primers to gain all possible types of RNA transcripts. Real-time PCR with primers designed to specifically recognize N- and C-terminus of β-galactosidase cDNA was used to amplify sequences at both 5′- and 3′-end of β-galactosidase RNA transcripts. We compared the amounts of full length and short abortive transcripts of β-galactosidase gene. The effect of FVP was dependent on the concentration. 25 nM FVP did not increase expression from either viral promoter (PCR-1 random hexamers) and decreased the quantity of β-galactosidase full length mRNA transcripts (PCR-2 oligo dT). Treatment by 100 nM FVP increased the expression from HIV-promoter (PCR-1 random hexamers) and the number of β-galactosidase full length mRNA transcripts in H1299-HIV cells (PCR-2 oligo dT). (TIF) [file pone.0089228.s003.tif]
